# Well-Being and the Risk of Depression under Stress

**DOI:** 10.1371/journal.pone.0067395

**Published:** 2013-07-01

**Authors:** Faren Grant, Constance Guille, Srijan Sen

**Affiliations:** 1 Department of Psychology, University of Maryland Baltimore County, Baltimore, Maryland, United States of America; 2 Department of Psychiatry, Medical University of South Carolina, Charleston, South Carolina, United States of America; 3 Department of Psychiatry, University of Michigan, Ann Arbor, Michigan, United States of America; Federal University of Rio de Janeiro, Brazil

## Abstract

Improving our ability to accurately predict individual risk for depression would have profound public health benefits. While there has been growing interest in understanding the relation between measures of positive emotion, such as well-being, and depression, it is not clear whether low well-being is an independent predictor of short term depression risk. We assessed whether low well-being is a risk factor for depressive symptoms. Medical internship is a well-established period of stress when levels of depressive symptoms increase dramatically. 1621 individuals beginning medical internship were assessed for well-being, depressive symptoms, and a set of psychological and demographic traits prior to starting internship year and again for depressive symptoms at 3 month intervals during the year. Low subjective well-being significantly predicted increased depression symptom scores during the stress of medical internship and accounted for individual level inter-variability in depression symptom trends across time. Assessing well-being may have utility in predicting future depression risk.

## Introduction

Major depression is characterized by a constellation of symptoms including sleep and eating disturbances, low mood, feelings of worthlessness, increased guilt, and suicidal ideation that affects approximately 16% of Americans at some point in life. Depression is the leading cause of lost worker productivity and days lost to disability [1], [2] and accounts for an annual capital loss of $36 billion [3]. Identifying early signs of individual-level depression risk has the potential to improve our ability to prevent depression and reduce the associated burden [4].

Most work on identifying depression risk factors has focused on demographic factors and negative risk factors such as adverse childhood environments, negative emotionality, and prior psychiatric history [5]–[7]. In recent years, some have suggested that positive psychological characteristics may be a protective factor for depression distinct from the absence of negative characteristics [8], [9]. Well-being is a characteristic of positive psychological functioning that captures an individual’s level of positive affect, life satisfaction, and sense of purpose in life [10]. Well-being has consistently been correlated with depression and other mental disorders in cross-sectional studies [11], [12], [13] but longitudinal studies are lacking. A ten year longitudinal study found well-being was predictive of internalizing mental disorders (i.e. depression, generalized anxiety disorder) [14]. The two longitudinal studies that have assessed the predictive value of well-being specifically on depression have had gaps of 10 [15] and 15 [16] years between assessments, limiting their applicability to clinical evaluations. In order to increase real-world clinical utility, it is important to assess if the absence of a positive characteristic such as low well-being can predict depressive symptoms across shorter time spans.

Unfortunately, because the natural incidence of new depressive episodes over short time periods is relatively low, designing studies to assess the short term predictive power of low well-being has been difficult. To determine if low subjective well-being is a predictor of depressive symptoms, we studied a cohort of individuals set to begin medical internship, a unique situation where a dramatic increase in depressive symptoms can be prospectively predicted. Medical internship is the first year of post medical school clinical training and is well-established as a period of high stress [17]. New physicians are faced with long work hours, sleep deprivation, loss of autonomy and extreme emotional situations [18]. In previous cohorts of the Intern Health Study, depressive symptoms increased from 4% prior to internship to a mean of 26% during internship [19]. Here, we take advantage of the medical internship model to determine whether adding a well-being assessment to previously identified risk factors improves our ability to predict changes in depressive symptoms. While controlling for other demographic and psychosocial factors, we hypothesize that low well-being prior to the internship year will be distinctly associated with higher levels of baseline depression symptoms across time.

## Methods

3202 incoming medical interns from internal medicine, general surgery, pediatrics, obstetrics/gynecology, emergency medicine, and psychiatry residency programs at 16 medical institutions and universities across the USA were recruited prior to the 2010–2011 and 2011–2012 medical internship years. Individuals in the participant pool were sent an email two months prior to starting internship and were invited to participate in the study. For 206 subjects, the invitation emails were returned as undeliverable and we were unable to acquire a valid email address. Of the remaining participants, 1621 participants agreed to take part in the study. For each medical internship year, surveys were administered at five time points: the baseline survey two months prior to the start of the internship year and interim surveys at months three, six, nine, and twelve of the internship year. All surveys were administered to participants through a secure online website.

### Ethics Statement

All conducted research was approved by the University of Michigan Institutional Review Board (IRB). Upon first entering the secure online survey, participants were shown the consent form. Participants were provided two options: clicking a yes button to acknowledge their consent to participate in the study or clicking a no button to decline participation in the study. Consenting participants were taken to the first page of the survey and declining participants were forwarded to a thank you page. A waiver of documentation of consent was approved by the University of Michigan IRB. All data was deidentified and no links between the identification number and the subjects’ identities were maintained by the researchers.

### Measures

The baseline survey assessed: 1) demographics (age, race/ethnicity, gender) 2) medical specialty 3) personal history of depression 4) family history of depression 5) depressive symptoms using the nine item Patient Health Questionnaire (PHQ-9) [20] 6) neuroticism levels using the NEO Five Factor Inventory [21] 6) the adversity level of the early family environment using the Risky Families Questionnaire [22] 7) average amount of alcohol consumed within the past month; 8) and well-being levels within the past two weeks using the Mental Health Continuum –Short Form (MHC-SF) [23]. Interim surveys assessed depressive symptoms using the PHQ-9. Well-being was additionally assessed at the six month survey.

### Mental Health Continuum

The Mental Health Continuum-Short Form (MHC-SF) is a 14 item measure of well-being that assesses three well-being subtypes: emotional well-being, psychological well-being, and social well-being. Items such as “how often do you feel happy”, “how often do you feel that your life has a sense of direction or meaning to it”, or “how often do you feel satisfied with life” are rated on a scale from 0 [never] to 6 [every day]. In parallel to diagnostic criteria for depression which assesses emotionality and functioning, the MHC-SF measures well-being across two domains: positive emotions and positive functioning. Items can be grouped based on scoring within the two domains yielding a categorization of flourishing (high positive functioning, high positive emotions) or languishing (low positive functioning, low positive emotions). Individuals scoring between flourishing and languishing are categorized as moderately mentally healthy. Additionally, items can be continuously scored. Subsequent analyses will use both the continuous and categorical scoring. The MHC-SF showed good internal consistency (>.80) and test-retest reliabilities of.68 over three months and.65 over nine months.

### Data Analysis

Growth curve modeling was utilized to assess changes in depressive symptom score trajectories over the internship year. For each individual participant, the growth curve model allowed for representation of the continuous outcome variable (depressive symptoms) as an individual growth trajectory. Individual growth trajectories are modeled as a function of time and the trajectories’ shapes are determined by an intercept (baseline depressive symptoms) and a slope (four repeated measures of depressive symptoms across the internship year). Growth curve models offer several advantages including the ability to analyze individual growth patterns for variables of interest across multiple points of time and robustness against missing data [24], [25].

The individual growth curve analysis was composed of two components. The first consisted of correctly specifying how depressive symptoms were changing over time across individuals. This specification included assessing for variability in intercept and slope and identifying the model’s form of growth. Model fit was evaluated by differences in −2 log likelihood (-2LL) scores between increasingly complex nested models. These statistics follow a chi-square distribution and mirror tests of change in chi-square. The second component examined which baseline variables predicted variability across individual depressive system trajectories.

Changes in PHQ-9 depressive symptom scores and well-being scores from baseline to medical internship were assessed using a paired t-test. SPSS version 19.0 (SPSS Inc, USA) was used for all analyses. The de-identified data will be made publicly available in the National Institutes of Health database of Genotypes and Phenotypes (NIH dbGaP) once genotyping is completed.

## Results

Descriptive statistics for our sample is listed in Table 1. In an earlier publication, we have shown that the subset of subjects who participated in our study was slightly younger (1.3 years old) and included a slightly higher percentage of women (1.3%) compared to the overall set of invited subjects. There were no significant differences in specialty, institution, or demographic variables between individuals who participated and those who did not [26].

**Table 1:** Sample Demographic Characteristics.

Category	Subcategory	Percentage
Age	≤25	17.7
	26–30	68.7
	31–35	9.4
	>35	1.5
Sex	Male	51.7
	Female	47.9
Marital Status	Single	58.1
	Engaged	9.6
	Married	25.9
	Other	0.8

Consistent with previous work, average PHQ-9 depressive symptom scores significantly increased from before internship (*M = *2.43, SD = 3.05) to 3 months (*M = *5.61, SD = 4.37; *t*(1140) = −26.90 *p*<0.001), 6 months (*M = *5.85, SD = 4.71; *t*(1096) = −26.08, *p*<0.001), 9 months (*M = *6.06, SD = 4.79; *t*(1014) = −26.06, *p*<0.001) and 12 months (*M = *5.47, SD = 4.50; *t*(958) = −22.19, *p*<0.001) of internship. Average well-being scores decreased significantly from before internship (*M = *54.41, SD = 11.51) to 6 months of internship (*M = *48.43, SD = 13.86), *t*(1017) = 16.03, *p*<0.001. Table 2 presents the means, standard deviations, and zero order correlations for the well-being predictors and depressive symptom scores.

**Table 2:** Means, Standard Deviations, and Zero-Order Correlations of Main Predictor Variables and Outcome Variables.

	1	2	3	4	5	6	7
1. Baseline depressive symptoms	1.00						
2. Wave 2 depressive symptoms	.44	1.00					
3. Wave 3 depressive symptoms	.40	.55	1.00				
4. Wave 4 depressive symptoms	.42	.53	.59	1.00			
5. Wave 5 depressive symptoms	.40	.53	.57	.61	1.00		
6. Baseline well-being score	−.46	−.33	−.35	−.35	−.29	1.00	
7. Baseline flourishing	−.37	−.28	−.27	−.28	−.25	.78	1.00
Mean	2.43	5.61	5.85	6.06	5.47	54.51	0.71
Standard Deviations	3.05	4.37	4.71	4.79	4.50	11.51	0.45

All correlations significant at *p*<.01.

Baseline flourishing variable coded as 0 = non-flourishing, 1 = flourishing.

**Figure 1:**
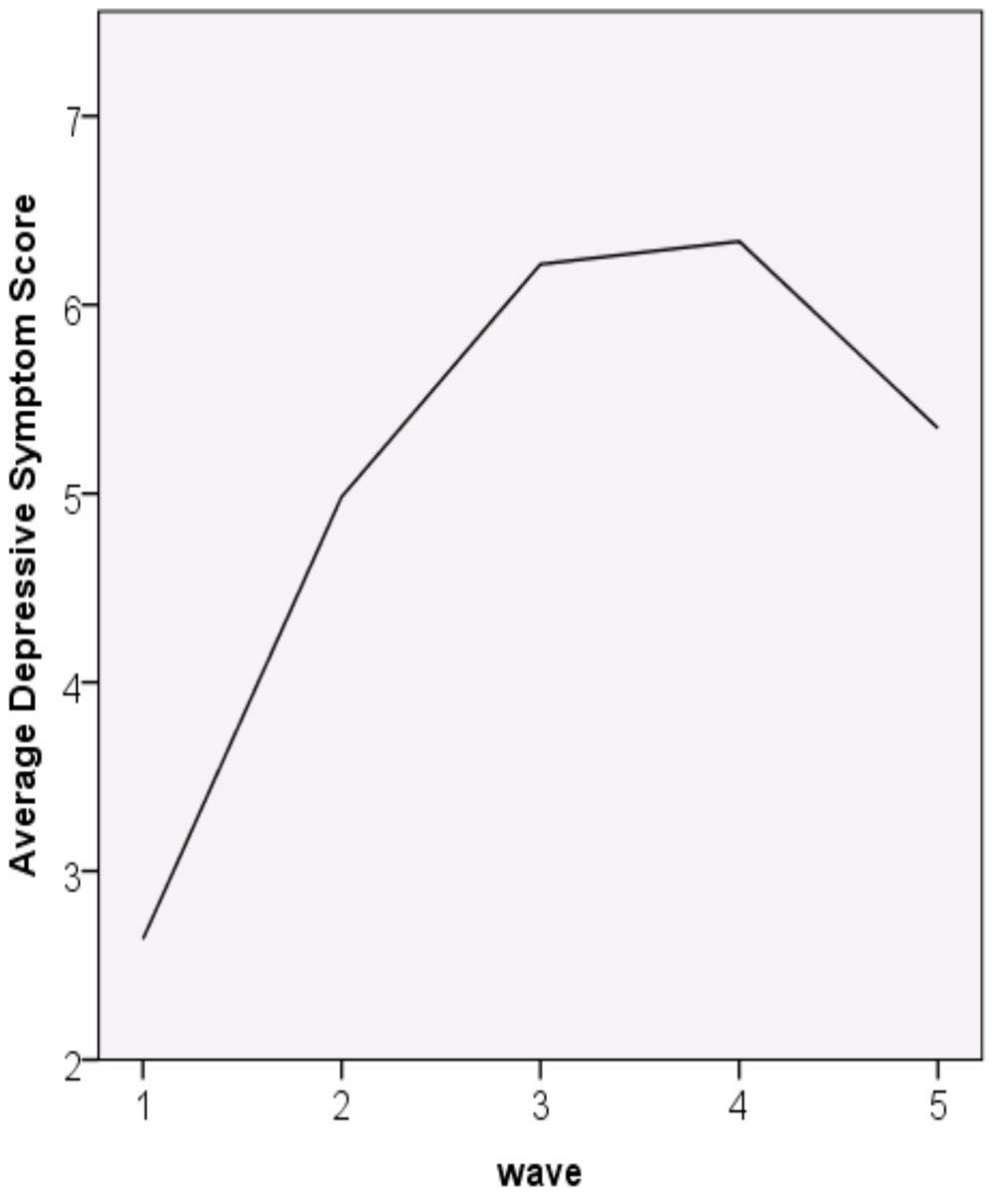
Average growth curve of depressive symptoms across the internship year.

Based on model fit information indicated in Table 2, we retained the fixed quadratic model. The fixed quadratic model indicates that individuals vary across their intercepts and slopes, that intercepts and slopes covary, and that the nature of the quadratic growth is the same across all individuals. The slope estimate was 2.90, *SE* = .09, *t*(4412) = 30.13, indicating that on average, individuals’ depressive symptom scores increased 2.90 units per measurement wave. The quadratic term estimate was −0.56, *SE* = .02, *t*(3961) = −23.55, suggesting an eventual downturn in depressive symptoms during the internship year. As figure 1 shows, there is an increase in depressive symptoms over most of the internship year with a slight downturn in symptoms at the twelve month survey.

**Figure 2:**
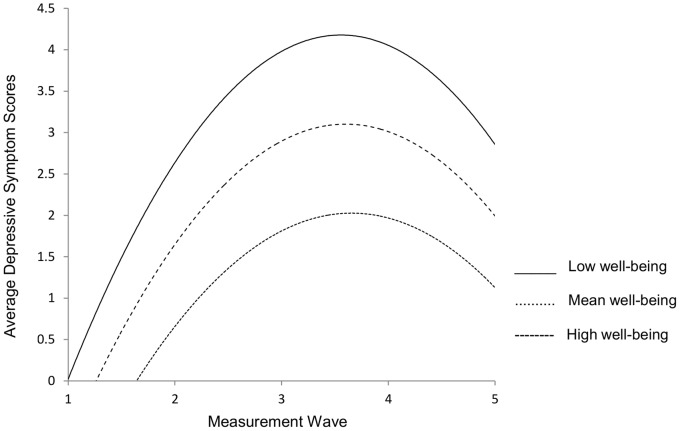
Growth curves of effects of high, mean, and low well-being on depressive symptoms.

After accounting for the baseline variables sex, race, age, personal history of depression, family history of depression, alcohol use, neuroticism, and early family environment, continuously measured well-being also accounted for individual level variability in depressive symptom score trajectories (Table 3). While well-being scores did not predict variability in intercepts, well-being did predict inter-individual differences in depressive symptom slopes and quadratic growth. As illustrated in figure 2, high well-being was on average associated with a 2.65 unit increase in depressive symptom slopes, mean well-being was on average associated with a 2.95 unit increase in depressive symptom scores, and low well-being was associated on average with a 3.25 unit increase in depressive symptom slopes. The estimates suggest that lower well-being scores before internship were associated with higher depressive symptom scores across the internship year.

**Table 3:** Model Fit Summary Information.

Model Name	−2 Log Likelihood (−2LL)	Change in −2LL (df)	*p*
Compound Symmetry	31902.33		
Unconditional Linear	31684.43	217.90(2)	<.001
Fixed Quadratic	31170.42	514.014(1)	<.001
Random Quadratic	34201.05	n.s	n.s

**Figure 3:**
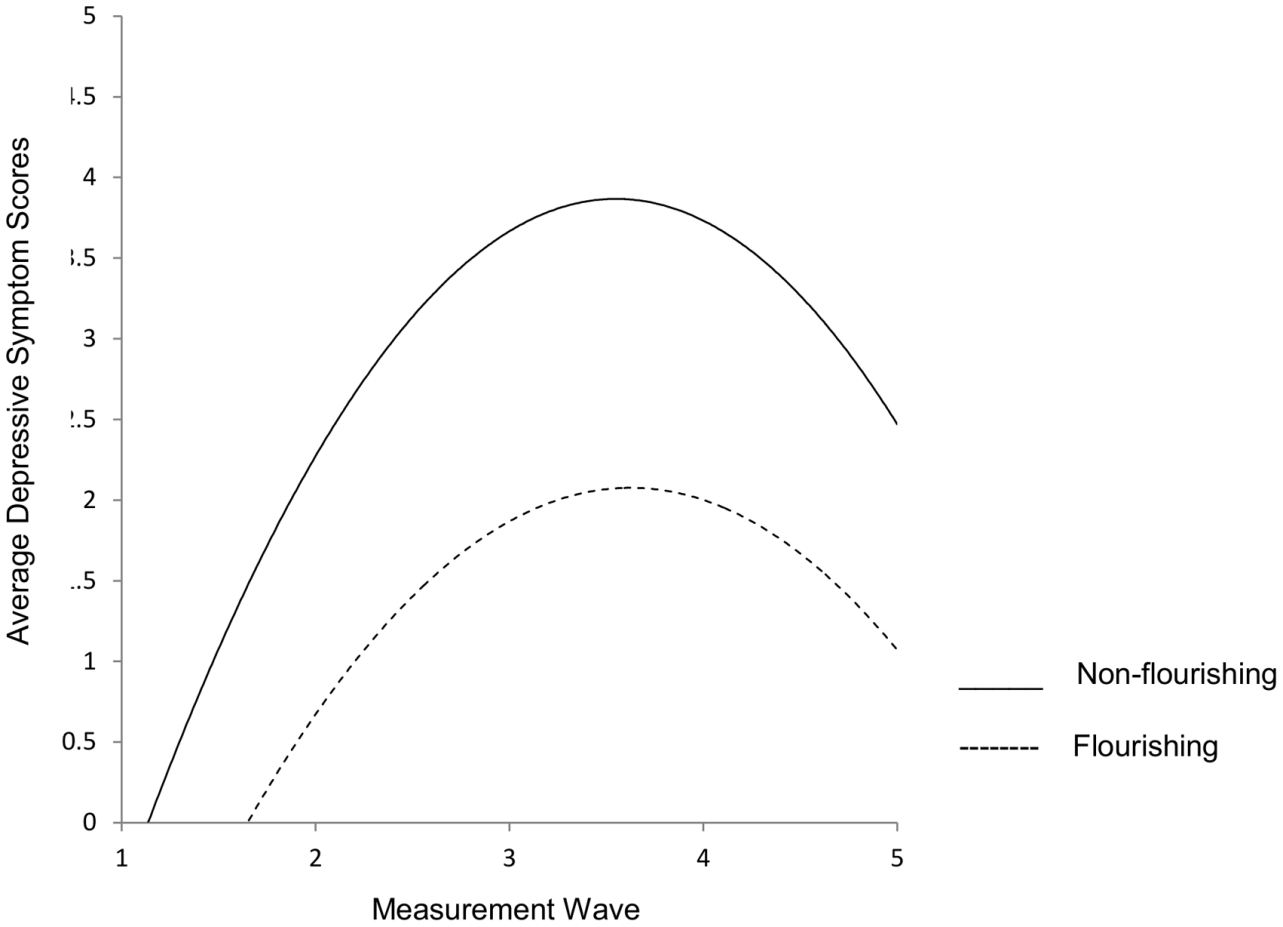
Growth curves of effects of flourishing and non-flourishing status on depressive symptoms.

As indicated in Tables 4 and 5, categorical scores of well-being also predicted depressive symptom slopes and quadratic growth. Compared to those who were flourishing at baseline, individuals who were languishing or moderately mentally healthy showed higher depressive symptoms across internship (Figure 3).

**Table 4:** Effect estimates for continuously measured well-being predicting depressive symptoms.

Well-BeingScore	Effects	Estimate	*SE*	*df*	*t*	*p*	95% CI
**High Well-** **being**	Slope	2.65	.13	4692	19.82	<.001	2.39–2.91
	Quadraticterm	−0.50	.03	4234	−15.17	<.001	−.56- −.43
**Mean Well-** **being**	Slope	2.95	.09	4674	31.17	<.001	2.76–3.14
	Quadraticterm	−0.57	.02	4193	−24.46	<.001	−.61- −.52
**Low Well-** **being**	Slope	3.25	.13	4725	24.19	<.001	2.99–3.51
	Quadraticterm	−0.64	.03	4286	−19.21	<.001	−.70- −.57

Well-being was centered with high well-being and low well-being calculated as 1 *SD* above and 1 *SD* below the mean, respectively.

**Table 5:** Effect estimates for categorically measured well-being predicting depressive symptoms.

Well-Being Category	Effects	Estimate	*SE*	*df*	*t*	*p*	95% CI
**Flourishing**	Slope	2.79	0.11	4580	24.98	<.001	2.57–3.01
	Quadratic term	−0.53	.03	4101	−19.43	<.001	−.59- −.48
**Non-flourishing**	Slope	3.38	.18	4633	18.77	<.001	3.03–3.74
	Quadratic term	−0.66	.04	4180	−14.98	<.001	−.75- −.58

Due to small size sample of languishing, non-flourishing included individuals categorized as both languishing and moderately mentally healthy.

## Discussion

Our results indicate that well-being distinctly predicts future depression risk. Specifically, we found that individuals who reported lower well-being at baseline showed significant increases in depression symptom scores across time. Well-being remained a significant predictor of depression score trajectories even after accounting for other baseline variables, such as neuroticism, early childhood environment, and gender that are established risk factors for depression. Importantly, low well-being remained a predictor of increased future depressive symptoms while also accounting for baseline depressive symptom scores.

These findings suggest that assessing well-being may add important practical utility to assessing for and preventing depression. Knowing that low well-being may increase the chances of developing depression could allow individuals and caregivers to take preventative steps before the onset of depressive symptoms [5]. Our findings also suggest that efforts specifically designed to increase well-being may be effective in protecting against depression and may aid in decreasing the overall health care costs associated with the disorder [12], [21], [27], [28]. This possibility should be assessed in future studies.

The study’s strengths were the large sample size of over 1500 participants, the ability to prospectively predict depression within the study design, and the use of repeated measures of depressive symptoms. There are limitations to our findings. First, our study was conducted on medical interns. While our intern sample was geographically and ethnically diverse, training physicians are a select population. Similarly, we detected our effect utilizing a specific stressor, medical internship. It is important to note that the other predictors of depression under internship (such as gender, neuroticism and an adverse early childhood) are confirmed predictors of depression risk in general. However, studies utilizing other populations and stressors should assess whether SWB is a predictor of depression under stress more generally. Next, although there were only modest baseline demographic differences in age and gender between those who chose to take part in the study and those who did not, only 53% of invited individuals chose to participate in the study. Finally, although the PHQ-9 instrument that we utilized to measure depressive symptoms has a sensitivity of 88% and a specificity of 88% for an MDD diagnosis with a cutoff of 10 points or higher [29], it is important to note that we assessed depression symptoms through a self-report inventory rather than a diagnostic interview.

In summary, we utilized the medical internship year as a model to assess the utility of well-being as a predictor of depression risk and changes in depression scores over time. We found that well-being, measured by a brief tool significantly predicted future levels of depression symptoms. These findings suggest that clinicians and researchers should assess well-being when they determine the risk for depression in their patients and participants.
